# OPRIPALC program: a regional perspective on pediatric radiation protection in interventional cardiology

**DOI:** 10.3389/fped.2026.1775615

**Published:** 2026-02-10

**Authors:** Carlos Ubeda, Eliseo Vañó, Patricia Miranda, Walter Mosquera

**Affiliations:** 1 Department of Medical Technology, LABODOP, Faculty of Health Sciences, Universidad de Tarapacá, Arica, Chile; 2 Radiology Department, Faculty of Medicine, Complutense University and IdIS, San Carlos Hospital, Madrid, Spain; 3 Dr. Luis Calvo Mackenna Children’s Hospital, Santiago, Chile; 4 ICESI University and Valle del Lili Foundation University Hospital, Cali, Colombia

**Keywords:** diagnostic reference levels, interventional cardiology, optimization, pediatric, quality assurance, safety culture

## 1 Introduction

Fluoroscopy-guided interventional procedures (FGIP) are now an essential component of modern pediatric care, particularly in interventional cardiology, where they have progressively replaced more invasive surgical approaches for the diagnosis and treatment of congenital heart diseases (1). These procedures offer clear clinical benefits, but they may also involve relatively high radiation doses. This raises specific concerns in pediatric patients, who are more radiosensitive and have a longer life expectancy, thereby increasing the potential risk of radiation induced effects later in life (2, 3).

From a radiological protection perspective, optimization of radiation protection exposure in pediatric interventional cardiology is therefore a priority. International organizations, including the International Commission on Radiological Protection (ICRP) (4), the World Health Organization (WHO), and the International Atomic Energy Agency (IAEA) (5), consistently emphasize the principles of justification and optimization in medical exposures. Within this framework, diagnostic reference levels (DRLs) are recognized as a key tool to support dose management while preserving clinical efficacy (6). In pediatric practice, DRLs are particularly important because they provide pragmatic benchmarks that help identify unusually high or low radiation doses and trigger local review and corrective actions.

Despite these recommendations, the implementation of DRLs in pediatric interventional cardiology has historically been limited and heterogeneous, outside of developed countries (6). Most published DRL data originate from single center experiences and show substantial variability in methodology, patient categorization, and reported dose metrics. This lack of harmonization has hindered meaningful comparisons and delayed the development of national or regional DRLs, particularly in less developed regions of the world (7–10).

To address these gaps, the Optimization of Protection in Pediatric Interventional Radiology in Latin America and the Caribbean (OPRIPALC) program was launched in 2018 as a joint initiative of the WHO, the Pan American Health Organization (PAHO), and the IAEA. OPRIPALC was conceived as a regional response to the need for structured optimization strategies in pediatric FGIP, with a particular focus on interventional cardiology. Beyond the establishment of regional DRLs, the program explicitly aimed to promote a culture of radiological protection, strengthen quality assurance (QA) practices, and foster multidisciplinary collaboration among pediatric cardiologists, medical physicists, and radiographers (medical technologists) (11, 12).

Since its inception, OPRIPALC has evolved into a large multicenter and multinational collaborative network. It has generated preliminary and updated regional DRLs for pediatric interventional cardiology and stimulated the development of local and national DRL initiatives within participating countries (13–17). At the same time, the implementation of such a complex regional program has revealed important limitations related to resources, technology, human capital, and sustainability (12, 18).

The aim of this Opinion article is to provide a critical appraisal of the strengths, weaknesses, and future opportunities of the OPRIPALC program, based on published evidence and documented regional experience. By reflecting on lessons learned, this work seeks to contribute to constructive discussion on how regional initiatives can effectively support optimization, QA, and safety culture in pediatric interventional cardiology.

## 2 Strengths

One of the principal strengths of OPRIPALC is its conception as the first coordinated regional initiative specifically focused on optimizing radiation protection in pediatric FGIP, particularly in interventional cardiology for countries of Latin America and the Caribbean. Prior to OPRIPALC, DRL initiatives in this field were largely limited to isolated single center studies (19–22). In contrast, OPRIPALC established a structured regional framework that enabled the derivation of harmonized regional DRLs across multiple countries in Latin America and the Caribbean. During the OPRIPALC program, 33 centers from 11 countries (Argentina, Brazil, Chile, Colombia, Costa Rica, Cuba, Ecuador, Mexico, Panama, Peru and Uruguay) participated. Of these, only 23 centers were able to send a total of 2,906 interventional cardiology procedures to establish the regional DRLs between December 2020 and December 2023 (12, 18).

A key methodological strength lies in the program's close alignment with international radiological protection recommendations. The use of the third quartile of patient dose distributions, stratification by age and weight bands, and the selection of kerma-area product as the primary DRL quantity are fully consistent with ICRP guidance. This methodological coherence ensures that the DRLs derived through OPRIPALC are scientifically robust, clinically meaningful, and directly comparable with those reported in other regions (6).

The most up-to-date regional DRLs, published in 2025 for different diagnostic and therapeutic procedures by age and weight groups for kerma-area product quantity, were 4.3–5.4 Gy cm^2^ (<1 year) to 20.6–34.8 Gy cm^2^ (10 to <16 years) and 3.4–4.5 Gy cm^2^ (< 5 kg) to 26.9–63.9 Gy cm^2^ (50-<80 kg), respectively. The proposed set of regional DRLs for *P*_ka_/weight values were 0.885–1.181 Gy cm^2^/kg (<1 year) to 0.510–0.838 Gy cm^2^/kg (10 to <16 years) and 0.984–1.333 Gy cm^2^/kg (< 5 kg) to 0.448–1.196 Gy cm^2^/kg (50-<80 kg), respectively (18).

Another major strength is the program's multidisciplinary approach. OPRIPALC actively integrates pediatric interventional cardiologists, medical physicists, and radiographers (medical technologists), recognizing that effective optimization cannot be achieved by any single professional group (23). In practice, radiographers play a central role in protocol selection, image acquisition, QA activities, and the routine collection of patient dose indicators. Their explicit inclusion has facilitated the translation of optimization principles into daily clinical practice and strengthened local ownership of radiation protection activities, especially in countries where the figure of the medical physicist specializing in interventional procedures is scarce.

Capacity building represents an additional strength. Through regular virtual meetings, regional workshops, and targeted training activities, OPRIPALC has supported continuous professional development in radiological protection and QA across participating countries. These efforts have improved awareness of pediatric specific radiation risks and promoted the gradual integration of QA programs in centers with diverse levels of resources (24).

Importantly, OPRIPALC has acted as a catalyst for local and national initiatives (25, 26). Several centers have adopted the OPRIPALC methodology to establish local DRLs, which subsequently serve as a foundation for national benchmarking (13–17). This adaptability and scalability make OPRIPALC potentially transferable model for other regions of the world.

## 3 Weaknesses and limitations

Despite its achievements, OPRIPALC has also revealed several structural and operational limitations. Differences in system generation, detector technology, and dose management produced by heterogeneity of x-ray equipment and imaging technologies across participating centers, capabilities inevitably influence dose metrics and image quality, limiting the degree of standardization achievable at a regional level.

A related limitation is the uneven availability of automatic dose management systems (DMSs). During the early phases of the program, dose data were often collected manually, increasing workload and the risk of incomplete or inaccurate data. Although progress has been made towards implementing automated solutions, DMS availability remains variable, restricting real-time monitoring and continuous quality improvement in some centers (27).

Human resource constraints also pose challenges. The availability of trained medical physicists and radiological protection professionals varies considerably across the region. In several centers, optimization and QA activities depend on a small number of individuals who often combine multiple roles. This can limit the frequency of dose audits and the systematic implementation of quality control programs.

Sustainability represents another concern. Participation in OPRIPALC is voluntary and depends on local leadership and institutional commitment. In the absence of binding regulatory requirements for pediatric DRLs in some countries, improvements achieved through the program may be vulnerable to changes in staffing, funding, or institutional priorities.

From a methodological perspective, regional DRLs inherently aggregate data from diverse clinical environments. While this provides valuable benchmarking, it may obscure differences in procedural complexity or case mix. Regional DRLs should therefore be interpreted as guidance values rather than performance targets and applied locally with appropriate professional judgement.

Finally, although OPRIPALC has focused primarily on radiation protection optimization, other dimensions of radiation protection, such as systematic assessment of occupational exposure, remain less developed and warrant further attention.

## 4 Opportunities and challenges

The experience gained through OPRIPALC offers clear opportunities for consolidation and expansion. Wider implementation of automatic DMSs would improve data quality, enable real time dose alerts, and support more effective quality improvement cycles. It would also allow more refined analysis of procedural complexity and dose trends.

Another important opportunity lies in translating regional DRLs into national reference frameworks. The successful establishment of local DRLs using the OPRIPALC methodology demonstrates its scalability. Building on these experiences, countries could progressively develop national DRLs aligned with their healthcare systems and regulatory contexts, with OPRIPALC serving as a technical backbone.

Proposing DRLs based on a nomenclature and levels of complexity standardized to the reality of Latin American and Caribbean countries represents a significant challenge, even though similar initiatives exist at the United States (28) and European levels (29).

Strengthening the role of medical physicists and radiographers represents a further priority. Structured training, certification, and professional recognition could enhance their contribution and reinforce multidisciplinary safety culture in pediatric catheterization laboratories.

Expanding OPRIPALC beyond pediatric interventional cardiology to other FGIP would further increase its clinical relevance and public health impact. From a research perspective, integrating patient and occupational dose monitoring would support a more comprehensive approach to radiation safety.

## 5 Discussion

The OPRIPALC program represents a meaningful advance in addressing persistent gaps in pediatric radiation protection in fluoroscopy-guided interventional cardiology in Latin America and the Caribbean. By combining regional coordination, harmonized methodology, and multidisciplinary engagement, the program has moved beyond isolated institutional initiatives and established a coherent framework for dose optimization that is both scientifically grounded and clinically relevant.

A central contribution of OPRIPALC is the demonstration that regional DRLs can be developed and applied in heterogeneous healthcare environments. The availability of preliminary and updated regional DRLs has provided participating centers with practical benchmarks against which local practice can be evaluated. Importantly, these values should not be interpreted as performance targets. In line with ICRP recommendations, DRLs must be understood as investigative tools that trigger critical review and local optimization actions (6). This distinction is essential to prevent inappropriate use of DRLs and to preserve their role as facilitators of quality improvement rather than constraints on clinical decision making.

The experience of OPRIPALC also reinforces the importance of multidisciplinary collaboration as a prerequisite for effective optimization. Pediatric interventional cardiologists, medical physicists, and radiographers contribute complementary expertise that directly influences radiation safety outcomes. The explicit inclusion and empowerment of radiographers and medical technologists has been particularly important, as it has enabled optimization principles to be translated into routine clinical practice. This approach supports the concept of radiation protection as a shared professional responsibility embedded within daily workflow.

At the same time, the limitations identified through OPRIPALC reflect broader structural challenges common to many world regions. Variability in equipment, uneven access to dose management systems, and shortages of specialized human resources constrain the pace and uniformity of implementation. Rather than undermining the value of the program, these challenges highlight the need for adaptive and pragmatic optimization strategies that can be progressively strengthened as resources and institutional support evolve.

Looking forward, the long-term impact of OPRIPALC will depend on its ability to transition from project based activities to sustained institutional and policy level integration. Alignment with national regulatory frameworks, professional society guidance, and QA requirements will be critical to ensure durability. Finally, beyond its regional context, OPRIPALC offers transferable lessons for other world regions settings facing similar constraints, contributing to the global effort to improve radiation safety and quality of care in pediatric interventional radiology and cardiology.

## 6 Conclusion

The OPRIPALC program demonstrates that meaningful advances in radiation protection in pediatric interventional cardiology can be achieved through regional coordination, even in heterogeneous and resource-constrained healthcare settings. By combining harmonized methodologies with multidisciplinary engagement, the program has provided practical reference points for radiation protection optimization while fostering a shared safety culture of radiological protection. Importantly, the experience confirms that regional diagnostic reference levels are most effective when used as investigative tools and adapted to local clinical practice rather than as prescriptive targets. The challenges identified, including variability in technology, limited human resources, and long-term sustainability, underscore the need for institutional commitment and alignment with national policies. Beyond Latin America and the Caribbean, OPRIPALC offers a pragmatic model for other regions seeking to strengthen radiation safety through collaboration, reinforcing the value of regional initiatives as drivers of quality improvement in pediatric interventional care.
